# Address

**Published:** 1867-01

**Authors:** A. Means


### ADDRESS.

### *A Voice from the Chemical Lecture Room of the Atlanta Medical College.*

To the survivors of 2,500 Medical Students, to whom it has been our privilege and pleasure to lecture during the last 26 years—800 of whom have, at various times, borne away from our Annual Commencement, the humble name of the writer upon their several Parchments of Graduation—this communication is respectfully and affectionately addressed.

It may not—nay, it will not meet the eye of *all* of them; but those to whom the medium through which it is presented may be accessible, will recognize in the signature appended, the name of an old and sincere friend of their scholastic years. Time, it is true, has not failed to leave its impress upon the physique of the Lecturer; but memory, busy memory, true to her task, brings up, in vivid reminiscence, the thousands of pleasant, and, he trusts, profitable hours, in which—surrounded by the paraphernalia of Science, and drafting heavily upon her laws—we canvassed together the wonders of the organic universe, in its chemical combinations and their compound results, traced the mysterious phenomina of life through the complicated mechanism of the human body, extending our researches through the labyrinths of Physiology, Pathology, and Therapeutics, that the inquisitive young minds, thus scientifically trained, might be amply panoplied for the professional responsibilities of after life.

But these halcyon days "are numbered with the years beyond the flood," to be known no more, and many of you have been since called to encounter the stern realities of a "co-minus" struggle with the adverse fortunes, the incorrigible prejudices, and the palpable ills, incident to your current career. Nay, some of you have grown gray, amid professional toils, since we met in the last grateful interview around the Laboratory table.

But still, amid the pauses of life's cares, you frequently and pleasantly recur to the years of your medical pupilage, and kindly remember the incidents and the friends of "auld lang syne." The Colleges of your early choice, however, still live to give annual paternity to your destined successors in the high walks of professional enterprise.

If, indeed, those to whom he addresses himself still feel an interest in the writer, and his connections with public life, they will benevolently tolerate the communicative view of the present article, in which, for the *first* time, he has ventured publicly to indulge, and may *never* indulge again. He still occupies the chair, then, of general and Medical Chemistry, in the Atlanta Medical College, with whose past history and modes of instruction many of you are experimentally acquainted. But its present status and popular claims, since its resuscitation from the paralyzing shock of war, demands some notice at our hands. It has held two sessions since its re-organization, and on the first of May next, will open its Halls—D. V.—for a regular didactic course of instruction; and with flattering prospects of success, will enter upon a more vigorous outlay of its energies, and an ample display of its resources. By the aid of a noble and generous appropriation of $5000, on the part of the City Council of Atlanta, the whole building and its surrounding enclosure have undergone thorough repairs: while its large and well ventilated Lecture and Dissecting Rooms, its pure atmosphere, and its delightful and refreshing water, whose mid-summer temperature is 58 degrees of Fahrenheit, furnish no inconsiderable attractions to medical students who consult their personal comfort while engaged in their pursuit of medical knowledge. Its suit of Chemical and Philosophical Apparatus, laid in under our own eye, and many of them manufactured to order, is one of the most magnificent and complete which can be found in any College, North or South. Two hundred dollars have been recently expended to make some select additions to the stock, and repair the injuries sustained by the war.

A Hospital has been built on the College grounds, in which, from 60 to 80 beds are constantly filled with the suffering poor of the country; and a regular Dispensary, established by the Faculty, furnishes from 15 to 20 cases daily, of such indigent sick as can come or be carried to the College for prescription and treatment in the presence of the class, and to which they have free and constant access. The facilities thus furnished, therefore, for the attainment of practical knowledge, adapted to the management of diseases peculiar to our Southern latitude, are already of a high order, and will continue to be increased, rather than diminished; while a full, competent, and experienced Faculty stand ready to do their whole duty in this important field.

The city, itself, has been re-built with unexampled rapidity, and now contains, probably, 5,000 inhabitants more than before it was dismantled and destroyed. Board is readily procurable, and abundant supplies of Drugs, Medicines, Books, and Instruments, are at the students' command, on terms as moderate as the times will justify. And here it may be proper to add, that the intelligent population of the place have ever extended a cordial welcome and the genial courtesies of life to the young gentlemen of the College.

In conclusion: While the writer would be greatly gratified to receive the kind consideration and patronage of his old students and friends, and greet a large and intellectual class at the approaching session, in furtherance of the prosperity of the College with which he is identified, yet he could only feel warranted in the expression of such a desire by a thorough conviction of its legitimate claims to public confidence, the high character of its medical instruction, the faithful and unsparing devotion of the Faculty to the interest and comfort of the student, and their fixed determination, ever to conform to the established code of medical ethics adopted by the associated wisdom of the profession, and within its appropriate sphere. Nor is he an exclusionist in his views. He encourages no invidious or ungenerous allusions to other kindred Institutions. They have their respective claims to an honorable and useful career, and a selfish and unscrupulous antagonism is below the dignity of the noble profession to which we all belong. With one of these Institutions he labored for 19 consecutive years, in the chair of Chemistry and Pharmacy. The members of its

Faculty were his dearest friends, and the disinterested kindness of some of them, in a time of deep affliction, and even since his withdrawal from their body, *will be cherished in life-long rememberance.* In the language of the immortal Webster, then, he "thanks God that if" he is "gifted with little of the spirit which is said to be able to raise mortals to the skies," he has "yet none," he trusts, "of that other spirit, which would drag Angels down." If the professional resources and intrinsic merits of the Atlanta Medical College, therefore, are unworthy to command public confidence and patronage, *let her fall.* But a nobler destiny, we believe to be hers, and with an abiding conviction of her work and rising prosperity, we cordially commend her to the support of a generous and enlightened public. An active and vigilant Board of Trustees throw the shield of their protection over all her external interests, and she launches afresh upon inviting seas.

A full Circular, containing all the particulars necessary to be known, preparatory to the opening of the approaching Term, on the first of May next, has been published and widely distributed.

With a deferential and respectful regard for the opinions of those to whom the foregoing communication is addressed, the writer subscribes himself,

Very truly, Theirs &c., A. MEANS.

